# Refraining from interaction can decrease fear of physical closeness during COVID-19

**DOI:** 10.1038/s41598-023-34667-x

**Published:** 2023-05-11

**Authors:** Katharina Kühne, M. A. Jeglinski-Mende

**Affiliations:** Cognitive Sciences Division, Department of Psychology, University of Potsdam, Karl-Liebknecht-Straße 24-25, House 14, Potsdam OT Golm, 14476 Potsdam, Germany

**Keywords:** Human behaviour, Health policy

## Abstract

Perception of peripersonal space (PPS) and interpersonal distance (IPD) has been shown to be modified by external factors such as perceived danger, the use of tools, and social factors. Especially in times of social distancing in the context of the COVID-19 pandemic, it is vital to study factors that modify PPS and IPD. The present work addresses the question of whether wearing a face mask as a protection tool and social interaction impact the perception of IPD. We tested estimated IPD in pictures at three distances: 50 cm, 90 cm, and 150 cm in both social interaction (shaking hands) and without interaction and when the two people in the pictures wore a face mask or not. Data from 60 subjects were analyzed in a linear mixed model (on both difference in distance estimation to the depicted distance and in absolute distance estimation) and in a 3 (distance: 50, 90, 150) × 2 (interaction: no interaction, shake hands), × 2 face mask (no mask, mask) rmANOVA on distance estimation difference. All analyses showed that at a distance of 50 and 90 cm, participants generally underestimated the IPD while at an IPD of 150 cm, participants overestimated the distance. This could be grounded in perceived danger and avoidance behavior at closer distances, while the wider distance between persons was not perceived as dangerous. Our findings at an IPD of 90 cm show that social interaction has the largest effect at the border of our PPS, while the face mask did not affect social interaction at either distance. In addition, the ANOVA results indicate that when no social interaction was displayed, participants felt less unsafe when depicted persons wore a face mask at distances of 90 and 150 cm. This shows that participants are on the one hand aware of the given safety measures and internalized them; on the other hand, that refraining from physical social interaction helps to get close to other persons.

## Introduction

### Protection measures in the framework of the COVID-19 pandemic

Following a two-year period of safety measures, our understanding of the world has been altered as a result of the COVID-19 outbreak. It is reasonable to assume that the implementation of protective measures, particularly social distancing regulations, has played a role in transforming our perception of ourselves and others.Social distancing as an important means to protect the population from infection has been applied in different ways in different societies. Australia, China, Denmark, France, Hong Kong, and Singapore, for instance, stuck to the interpersonal distance (IPD) suggested by the World Health Organization, i.e., at least 1 m or 3 feet. Other countries extended the recommended IPD to 2 m (Brazil), to 1.8 m (the United States), or 1.5 m (Germany, Belgium, Italy, and Portugal, among other European countries). Additionally, another protective measure during the pandemic was until recently the recommendation to wear a face mask. Before the COVID-19 pandemic, wearing face masks was not a normal habit for most of the population, with some exceptions, such as medical professionals.

Further safety recommendations suggested having as little physical contact with others as possible. Thus, shaking hands became a taboo for many of us or was immediately followed by cleaning hands. Normal, polite social interaction became unusual in the last 2 years. On these grounds, one can assume that the aforementioned protective measures changed our natural behavior both concerning proxemics (distancing), perception of others (wearing a face mask), and willingness to physically interact (danger of social interaction).

Importantly, adherence to the protective measures can be impacted by mental health, that, in its turn, can be influenced by other safety measures, such as a lockdown. For example, several studies conducted in Vietnam at the beginning of the pandemic showed that a partial lockdown increased levels of depression, stress, and anxiety, especially among single, separated, or widowed individuals, individuals with a higher education level, and a larger family size. Other factors making individuals especially vulnerable were job loss and having contact with potential COVID-19 patients^1^. Less outdoor activity and fear of being infected were possible reasons for the increased level of depression and anxiety among Vietnamese citizens^2^. Similar results were reported from China^3^ and the USA^4^. In the same vein, the pandemic was associated with increased burnout levels among Hong Kong citizens^5,6^. On the other side, other protective factors such as hand hygiene, wearing a mask, and confidence in medicine reduced the psychological impact of the pandemic^3^. Also in resuming in-office work after lockdown psycho-neuroimmunity prevention measures such as masks proved to be effective in reducing stress^7^. Wearing a face mask at the community level was one of the major factors improving not only physical but also mental health, as a cross-country comparison study between Polish and Chinese participants showed^8^. Thus, there was a complex interplay between safety measures, mental health, and social behavior. The present study aims to research the comfortable IPD in the context of the COVID-19 pandemic with respect to social interaction and wearing face masks. To motivate our research questions, the next paragraphs provide a short overview of the relevant findings.

### The peripersonal space and the comfortable interpersonal distance in the context of the COVID-19 pandemic

The term peripersonal space (PPS) was first introduced by Rizzolatti et al.^9^ and describes the space around the body in which we escape threats, reach objects and prepare actions. Originally it was assumed to be a rather static area of 30–50 cm around the body and 60 cm around the head^10^. Nonetheless, research has shown that the PPS is flexibly modified by factors such as tool use^11–13^, the presence of another individual^14^, pregnancy^15^, environment temperature^16^, pressure^17^, hormone administration^18^, and reward^19^. Moreover, negative stimuli, i.e., threats, near our body change the representation of the PPS because the perceived reaching range changes^20–22^.

Similarly, the IPD also is perceived as flexible and can be changed by various factors, such as cultural conventions^23^, the degree of cooperation with another individual, age, and gender^24^. Nonetheless in German society, the general comfortable IPD is estimated to be about 90 cm^25^. Already in the 1960s, Argyle and Dean^26^ postulated that the comfortable IPD includes both positive (approach) and negative (avoidance) forces. The comfortable IPD reduces in cooperation as well as in safe or comfortable situations and increases in threatening or uncomfortable situations^23,27–29^, when we perceive negative emotions, such as arousal^30^, contamination danger^31,32^, fear of contagious diseases^33^, and social threat^34^. At the same time, discomfort has been reported when interacting at larger distances^35^.

PPS and IPD are closely interrelated, and both are flexibly adaptable. Thus, when the PPS changes, our comfortable IPD is also affected^36^. Nonetheless, one can distinguish between these two concepts when keeping in mind that the PPS describes rather the reachability to other individuals or objects while the IPD refers to the feeling of comfortable distance to other individuals^28^.

In the last few years, several research projects have assessed social distancing and its relation to PPS, IPD, perception of danger, and human approach behavior in general. These relations also might have changed as protective measures during the COVID-19 pandemic were introduced.

At the beginning of the COVID-19 pandemic, a study conducted in Switzerland documented a reduction of PPS due to social distancing^37^. Participants were asked to rapidly respond to a tactile stimulus in their faces in two conditions. In the first condition, participants reacted only to tactile stimulation (tactile, unimodal). In the second condition, participants were shown an approaching avatar in a virtual reality environment while they reacted to the simulation (visuotactile, multimodal). The authors used five distances: D1 ≈ 45 cm, D2 ≈ 80 cm; D3 ≈ 115 cm; D4 ≈ 150 cm, and D5 ≈ 185 cm. Interestingly, in the participants that were recorded before and within the first lockdown period (before June 10, 2020), reaction times were significantly faster in the multimodal condition in the closer distances (in distances 1–4, but not in the farthest distances = distance 5). On the contrary, after the first lockdown period (June 10–July 25, 2020), shown avatars did not elicit this facilitation when they were displayed farther away (the difference was only significant at the closest distance). Contrary to what one would expect, these findings suggested that PPS reduced after the lockdown, which was indicated by the lack of facilitation. The authors argued that after the lockdown the gradient of differentiation between the own PPS and the space of other individuals became sharper. Consequently, other individuals did not trigger any anticipatory response when they were far away, as before the lockdown. Rather, they were processed more strongly when they were at a close distance and provided a potential risk for contamination. An interview study showed that in Germany, the comfortable IPD enlarged during the pandemic: Participants reported that given the infection risk, they wanted to keep a greater distance from other people and anticipated keeping this larger IPD even after the pandemic has ended^35^. Crucially, this reported willingness of keeping an enlarged IPD during the pandemic was not in line with the at that time ongoing protests against the urge to wear face masks and to follow social distancing rules^38^.

As mentioned above, threatening stimuli might increase the PPS and the comfortable IPD while positive stimuli possibly have the opposite effect. In the context of the pandemic, a face mask could signal both danger (contamination risk) and safety (protection from the virus). Consequently, if it signals protection, a face mask possibly enlarges our PPS^39^, and assumingly, associated with it, the IPD. For instance, it was shown that test subjects had a larger comfortable IPD to a person who was wearing a face mask than a person without a face mask^40,41^. The face mask induced avoidance behavior by inducing a strong feeling of contamination risk both studies were conducted at the beginning of the pandemic when the application of face masks was still unusual.

On the contrary, in an online study with French subjects, a face mask reduced IPD because it signaled trustworthiness^42^. Crucially, the effect was higher in participants who were or had been infected with COVID-19. This effect might be caused by a feeling of safety related to assumed immunity after infection. On the other hand, a previous infection might have been caused by a priori general willingness to take a shorter distance to other individuals. This effect was replicated in males while in female participants, the emotional expression of a depicted face was the primary factor for taking a comfortable interpersonal distance, with shorter distances for happy faces than for angry ones^43^.

In this vein, Lisi et al.^44^ found that the comfortable IPD was reduced towards individuals who wear protective equipment in general or did not suffer from COVID-19 (proven with a negative COVID test).

The above-mentioned studies showed that both PPS and IPD are modified by external factors, i.e., the use of a face mask, infection of another person, or facial expressions. Moreover, contamination of the self and others apparently influences PPS and IPD as well. During the pandemic, the perception of face masks changed from signaling danger to signaling protection (reflected by the mixed results of previous studies^40–44^). We rely on social signals, especially facial expressions, to regulate an appropriate IPD^45,46^, which becomes challenging when the other person is wearing a face mask. Research found that standard masks interfere with emotion recognition and trust attribution^47^, recognition^48^ and, most importantly in the context of our research, reduce accuracy in the perception of closeness^49^. However, a recent study using Augmented Reality found that participants were quite accurate in estimation when judging the distance of avatars with and without a face mask and in action (coughing) or static^50^. Note that in all aforementioned studies, the modulations of PPS and IPD are probably not caused by real danger but by the individual perception of risk^51^.

### The present study

The present study aimed to investigate the impact of face masks and social interaction on the perceived IPD of others. Specifically, the study sought to determine whether face masks were perceived as a safety cue or a risk, and how social interaction and real-world distances affected perceived IPD.

Crucially, in our study, we did not explicitly ask participants to estimate their own comfortable IPD to another person^40,41^, a virtual avatar^37^, or a face^44^. Instead, we used a self-created distance classification task where participants were asked to estimate the distance between two depicted individuals by typing the estimated absolute distance in a free-text field for each stimulus. We used pictures that contained two persons standing next to each other at three distances (50 cm, 90 cm, and 150 cm). The three distances were developed as they would be if the persons stood in front of each other in a room. These distances were proportional to the body size of the persons depicted in the image. The individials in the pictures either were socially interacting (shaking hands or were preparing to interact by reaching out to shake hands) or were not interacting. Additionally, the persons in the pictures either wore a face mask or not.

By asking participants to explicitly provide a numerical estimate of the IPD between the individuals in the picture from a third-person perspective, the perceived comfortable IPD and risk were implicitly assessed. Measuring a comfortable IPD from a real-world first-person perspective was not possible at the moment due to safety restrictions and contamination risk. In our previous study, a similar paradigm was utilized, but with reaction times (RTs) as the dependent variable instead of distance estimation^52^. The same stimuli were used, and participants were asked to respond to the stimuli with a button press. We found that observers reacted fastest, indicating that they showed the least avoidance, for the shortest distances (50 and 90 cm) when individuals wore a face mask and did not interact, a face mask serving as a visual cue for safety.

Even though the subjects were not themselves displayed in the pictures, mirroring mechanisms would provide insights into their own comfortable IPD: Previous studies have shown that the human brain shares common mechanisms when we perceive actions performed by others and when we act ourselves^53,54^. Additionally, a recent study demonstrated that social distancing rules have become deeply embedded in our cognitive system and that we may even project these rules onto visual stimuli that do not necessarily relate to social distancing, e. g. logos^55,56^.

We predicted that the closer the depicted persons were, the more the participants would underestimate the distance between them (H1), reflecting an avoidance intention due to the perceived risk closeness provides in the context of the pandemic (in line with Marchiori^40^ and Seres et al.^41^ who found such effect for the subjects’ comfortable IPD; also, in line with Cartaud et al.^42^ Calbi et al.^43^, Sakuma and Ikeda^57^, and Welsch et al.^35^ who found similar effects in prolonged reaction times). Further, we expected an underestimation of depicted real-world distance in pictures where social interaction was displayed compared to pictures without social interaction (H2) due to the inappropriateness and danger of physical interactions in the context of the pandemic. Moreover, we predicted underestimation in pictures where depicted persons do not wear a face mask and overestimation in pictures where they wear a face mask (H3), caused by avoidance behavior and perceived danger.

## Material and methods

### Participants

One hundred and twenty-eight participants (113 native German speakers, 15 participants with another native language and self-reported fluency in German; 44 male, 79 female, 3 non-binary; 2 unspecified; mean age = 25 years and 4 months, SD = 7 years; 39.84% with university qualification of at least a bachelor’s degree; 113 right-handed, 13 left-handed; 2 ambidextrous) were tested in an online study hosted on Gorilla^58^. Participants were recruited at the University of Potsdam via SONA systems and received course credit for their participation.

Data were recorded from December 2021 to March 2022. In this period, there had been already several peaks of the pandemic wave in Germany, and safety guidelines had been implemented in society for months, so we assumed that participants were already used to safety rules and social distancing.

The study was conducted in accordance with the ethical guidelines that were laid down in the Declaration of Helsinki^59^. Since we used standard methods, did not expose the participants to any risk, and obtained participants’ informed consent before the study no further ethical assessment was needed. Further, the procedure was previously evaluated by professional psychologists to be consistent with the ethical standards of the German Research Foundation (DFG). Before the study started, participants were instructed about the task, read a data-protection statement of the University of Potsdam, and were informed about their rights as participants. Then, they gave their informed consent by ticking a box. Without giving consent, participation was not possible. There is confirmation from the Vice Dean for Research at the University of Potsdam that a full review of the study protocol by the institutional ethics committee was not required.

### Task and stimuli

Participants were told that the study measured estimated distances and were asked to spontaneously estimate the absolute real-life distance between two people displayed in the pictures as fast and as correctly as possible. Pictures were shown for 3000 ms to prevent participants from using measuring devices on their screens and to obtain spontaneous answers. Answers were given by typing a numerical value into a free-text field below the respective picture. The stimuli were shown in a randomized order. We did not indicate a specific body part of the depicted persons for the measure not to bias the results by leading the participants’ attention to the face masks or the hands. In addition, we did not specify a certain range of possible answers.

For stimuli, we used self-created pictures that were made from license-free and licensed stock images from internet databases. Licensed images came from the databases Adobestock, Shutterstock, and 123rf. We used pictures of persons that were wearing neutral or business-like clothes. We created the stimuli by cutting the background from the pictures so that we were able to combine the two persons into one picture using MS PowerPoint and GIMP. In the next step, we combined the persons in the pictures in such a way that the real-world distance between them would be about 50 cm, 90 cm, or 150 cm (a near, middle, and far distance, respectively). We used the body size of the depicted persons as a reference for creating the distances. Moreover, we manipulated the interaction between the depicted persons, i.e., they were either not interacting or were shaking hands/were preparing to shake hands. Finally, they were either wearing a face mask or not. We used a light-blue standard face mask and copied it in the respective pictures to create the illusion that persons wore a face mask.

**Figure 1:**
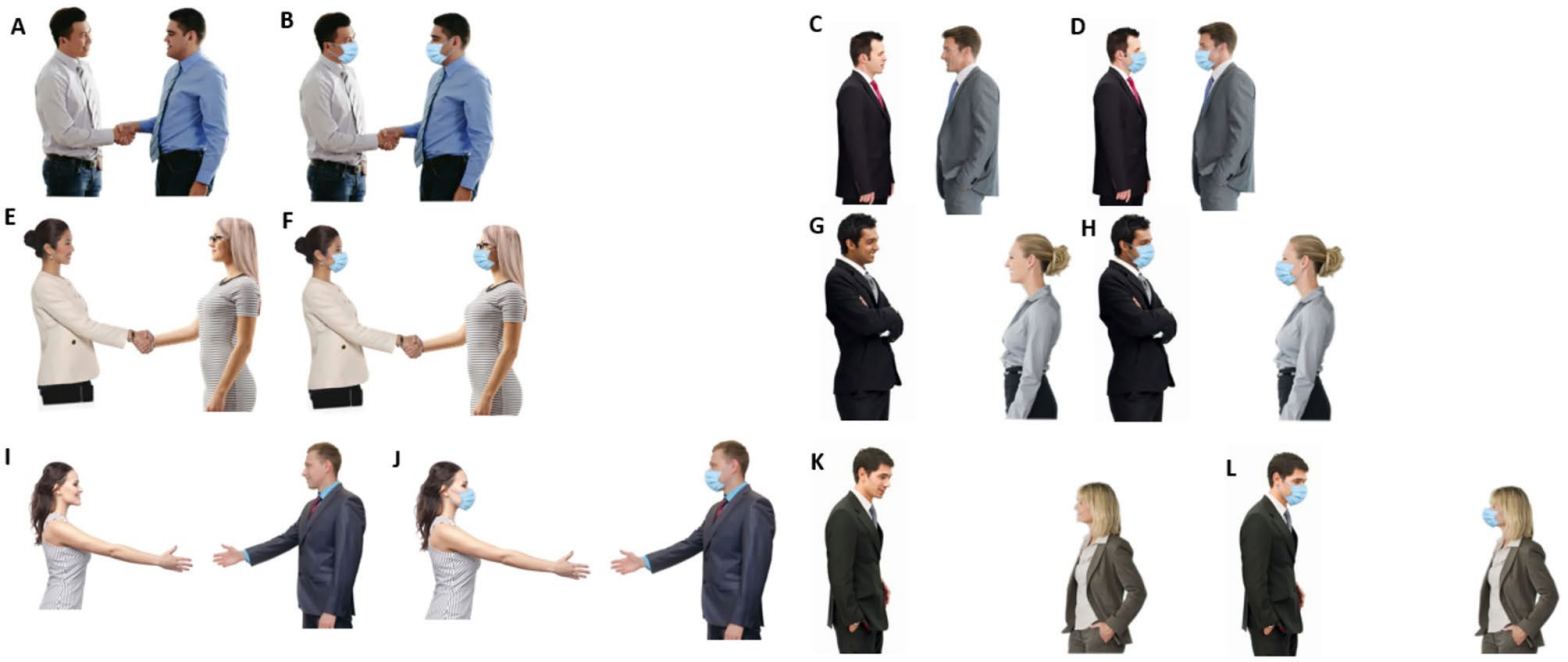
Selected stimuli of the experiment. (**A**): Male-male, 50 cm, interaction, no mask; (**B**): male-male, 50 cm, interaction, mask; (**C**): male-male, 50 cm, no interaction, no mask; (**D**): male-male, 50 cm, no interaction, mask; (**E**): female-female, 90 cm, interaction, no mask; (**F**): female-female, 90 cm, interaction, mask; (**G**): male–female, 90 cm, no interaction, no mask; (**H**): male–female, 90 cm, no interaction, mask; (**I**): female-male, 150 cm, interaction, no mask; (**J**): female-male, 150 cm, interaction, mask; (**K**): male–female, 150 cm, no interaction, no mask; (**L**): male–female, 150 cm, no interaction, mask.

Stimuli contained different gender combinations, i.e., female-female, female-male (female on the left), male–female (female on the right,), and male-male pairs. This combination of features led to 48 different stimuli with 3 levels of distance (near; middle; far), 2 levels of interaction (no interaction; shaking hands), 2 levels of face mask (wearing a face mask, no face mask), 4 levels of gender (female-female; female-male; male–female; male-male). Within each gender, distance, and interaction condition, the pairs of depicted persons were shown twice, once with a face mask and once without a face mask. Each participant saw each picture once. Sample pictures are depicted in Fig. 1. See Appendix I on OSF (https://osf.io/dsr86/) for a full list of stimuli.

The main dependent variable was the difference between the estimated distance and the actual distance between the depicted persons. Another dependent variable was the absolute distance between the depicted persons estimated by the participants (see also below, “Analysis and results”). We calculated the distance difference by subtracting the actual distance from the estimated distance. If this value was positive, participants overestimated the distance between the depicted persons, if it was negative, they underestimated this distance.

After the main experiment, participants answered demographic questions, a questionnaire about their handedness (Edinburgh handedness inventory–short form^60^), the General Anxiety Disorder Scale^61^ (GAD-7), and questions about the COVID-19 pandemic. In the General Anxiety Disorder Scale questionnaire, participants were asked to rate their symptoms over the past two weeks, e.g., trouble relaxing, feeling nervous, or becoming easily annoyed on a 4-point scale (“not at all”, “several days”, “more than half the days” and “nearly every day”).

Questions about the COVID-19 pandemic were:

1. How dangerous do you consider COVID-19 for yourself? (*Als wie gefährlich schätzen Sie die COVID-19-Pandemie für ****sich**** ein?* On a Likert scale from 1 = completely undangerous to 5 = very dangerous);
2. Do you consider COVID-19 as a danger for other people? (*Schätzen Sie die COVID-19-Pandemie als Gefahr für ****andere**** Menschen ein?* On a Likert scale from 1 = completely undangerous to 5 = very dangerous);
3. Do you consider COVID-19 as a danger for young people? (*Schätzen Sie die COVID-19-Pandemie als Gefahr für ****junge**** Menschen ein?* On a Likert scale from 1 = completely undangerous to 5 = very dangerous);
4. Do you consider COVID-19 as a danger for old people? (*Schätzen Sie die COVID-19-Pandemie als Gefahr für ****ältere**** Menschen ein?* On a Likert scale from 1 = completely undangerous to 5 = very dangerous);
5. Do you think that wearing a face mask can protect from COVID-19? (*Sind sie der Meinung, dass das Tragen von Atemschutzmasken Sicherheit vor einer Ansteckung bietet?* On a Likert scale from 1 = no protection at all to 5 = very good protection);
6. Do you live together with someone who has risk factors for COVID-19? (*Wohnen Sie mit jemandem zusammen, der Risikofaktoren für eine COVID-19-Infektion hat?* Yes/No);
7. Do you live together with someone who has been or is infected with COVID-19 (at the moment or before)? (*Wohnen Sie mit jemandem zusammen, der sich mit COVID-19 infiziert hat (momentan oder in der Vergangenheit)?* Yes/No);
8. Do you have risk factors for COVID-19? (*Haben Sie Risikofaktoren für eine COVID-19-Infektion?* Yes/No);
9. Have you been or are you infected with COVID-19 (at the moment or before)? (*Haben Sie sich schon mal mit COVID-19 infiziert (momentan oder in der Vergangenheit)?* Yes/No);
10. Are you worried that you might get infected with COVID-19? (*Befürchten Sie, sich mit COVID-19 zu infizieren?* On a Likert scale from 1 = not at all at all to 5 = very much).

After answering the questions participants were thanked for their participation and debriefed.

### Ethics statement

The study was conducted in accordance with the guidelines laid down in the Declaration of Helsinki. The methods were standard, there was no risk, participants gave their informed consent, and the procedure was already evaluated by professional psychologists to be consistent with ethical standards of the German Research Foundation (DFG), including written informed consent and confidentiality of data as well as personal conduct. There is confirmation from the Vice Dean for Research at the University of Potsdam Prof. Dr. Martin H. Fischer that a full review of the study protocol by the institutional ethics committee was not required.

## Analysis and results

Data preprocessing was conducted using MS Excel (Microsoft 365). First, a reality check was performed. Our expectation interval went from 25 cm (half of the near distance) to 300 cm (double of the far distance). If participants answered outside these borders in more than 20% of the trials, their data were not further analyzed (n = 65). In addition, three subjects were discarded because they stated not having seriously fulfilled the task. The remaining data (2,880 trials) were further analyzed.

### Linear mixed model analysis on the difference in the estimated distance

Participants whose data was included were overall very close and exact in their estimations in the overall testing: The grand mean of all estimations difference was almost 0 (in line with the results of Chakraborty et al.^50^).

We conducted linear mixed model analysis in R (Version 1.4.1106) using the lme4 package55^62^. We included the difference in the estimated distance (mean-centered) as the dependent variable and added fixed effects of face mask, interaction, living with someone infected with COVID-19, living with someone with COVID-19 risk, own infection, own risk, gender, face mask as protection, fear of COVID-19, danger for others, danger for the elderly, danger for young people, danger for oneself, screen size, and age. We included participants and stimuli as random effects (only intercepts for parsimony reasons). We removed two participants because of missing values in the gender variable (one stated their gender as “other” and another participant did not state it at all).

Categorical predictors (face mask, social interaction, living with someone infected with COVID-19, living with someone with COVID-19 risk, own infection, own risk, gender) were sum-coded. Categorical predictor distance with three levels was coded with successive differences contrast coding. Continuous predictors (mask as protection, fear of COVID-19, danger for others, danger for the elderly, danger for young people, danger for oneself, screen size, age) were coded in a continuous manner. We further conducted a backward elimination using the drop1 function to identify the best-fit model; Effects that did not improve the model fit (*p* > 0.1) were successively eliminated. In total, the fixed effects explained around 21% of the total variance. The mean random effect variance was 38% out of the total 51% of the variance explained by the model (31%).

The main effect of distance was significant: (*b* = -5.63 *p* < 0.05 and *b* = 25.83, respectively, *p* < 0.001). Comparatively 90 cm to 50 cm the distance was underestimated, and comparatively from 150 to 90 cm was overestimated (H1 partially confirmed). The main effect of social interaction was also significant, the participants underestimated the distance when social interaction was depicted (*b* = − 14.13, *p* < 0.001) (H2 confirmed). The main effect of a face mask was not significant (*b* = 1.98, *p* = 0.394) (H3 rejected). The interaction between face mask, distance, and social interaction was not significant (*b* = -11.46, *p* = 0.314 and *b* = 11.40 respectively, *p* = 0.317).

Two control variables reached significance: living with someone with a risk for infection with COVID-19 (*b* = 10.39, *p* < 0.05) and previous own infection with COVID-19 (*b* = 10.16, *p* < 0.05). Those who lived with someone having a risk of COVID-19 and participants who had been infected rather overestimated the distance between the individuals.

The following factors did not reach the level of significance in the model: living with someone infected with COVID-19, age, own risk, gender, fear of COVID-19, mask as protection, danger for oneself, danger for the elderly, danger for others, danger for young people, and GAD-7. Results are summarized in Table 1.

**Table 1:** Results of the linear mixed model analysis on difference estimates.

Predictors	Difference (centered)			
	Estimates	Std. Error	Statistic	p
(Intercept)	4.294520	2.871977	1.495318	0.135
Distance2-1	− 5.632861**	2.845079	− 1.979861	**0.048**
Distance3-2	25.829959***	2.847164	9.072171	** < 0.001**
Interaction	− 14.129390***	2.325002	− 6.077153	** < 0.001**
Face mask	1.981377	2.324897	0.852243	0.394
Covid live risk	10.385111**	4.942765	2.101073	**0.036**
Own infection	10.160805**	4.930931	2.060626	**0.039**
Risk other c	6.372543*	3.305271	1.927994	0.054
Distance2-1*Interaction	− 11.579402**	5.689812	− 2.035112	**0.042**
Distance3-2*Interaction	− 6.183128	5.694422	− 1.085822	0.278
Distance2-1*Face mask	4.571759	5.689886	0.803489	0.422
Distance3-2*Face mask	− 1.152180	5.694035	− 0.202349	0.840
Interaction*Face mask	− 7.356908	4.649828	− 1.582189	0.114
Distance2-1*Interaction*Face mask	− 11.458153	11.379848	− 1.006881	0.314
Distance3-2*Interaction*Face mask	11.397314	11.387947	1.000823	0.317
Random effects				
σ^2^	550.407738			
τ_00 id_	278.359867			
τ_00 Stimulus_	55.028085			
ICC	0.377223			
N_id_	58			
N_Stimulus_	48			
Observations	2693			
Marginal R^2^/conditional R^2^	0.214/0.510			

Note. Diff.c ~ Distance * Interaction * Face mask + Covid_live_risk + Own_infection + Risk_other.c + (1|id) + (1|Stimulus)* p < 0.1 ** p < 0.05 *** p < 0.01.

Significant values are in [bold].

### Linear mixed model analysis on the absolute estimated distance

We further conducted another linear mixed model analysis where we included the estimated distance (mean-centered) as the dependent variable and again added fixed effects of face mask, social interaction, living with someone infected with COVID-19, living with someone with COVID-19 risk, own infection, own risk, gender, face mask as protection, fear of COVID-19, danger for others, danger for the elderly, danger for young people, danger for oneself, screen size, and age. We included participants and stimuli as random effects (only intercepts for parsimony reasons). Variables were coded in the same way as in model 1. Here, the fixed effects explained around 75% of the total variance (84%). The mean random effect variance was 38%.

The main effect of distance was significant: (*b* = 34.37 and *b* = 85.83, respectively, *p* < 0.001). Comparatively 90–50 cm and from 150 to 90 the distance estimation was larger, which is trivial. The effect of social interaction was also significant (*b* = − 14.13, *p* < 0.001), there was more underestimation with interaction. Living with someone with COVID-19 risk (*b* = 10.39, *p* < 0.05) and own infection (*b* = 10.16, *p* < 0.05) had a significant effect.

The interaction between face mask, distance, and social interaction was not significant (*b* = − 11.46, *p* = 0.314 and *b* = 11.40, *p* = 0.317, respectively). Table 2 provides a summary of the results.

**Table 2:** Results of the linear mixed model analysis on absolute estimates*.*

Predictors	Estimation (centered)			
	Estimates	Std. error	Statistic	p
(Intercept)	4.646152	2.871976	1.617755	0.106
Distance2-1	34.367139***	2.845079	12.079503	** < 0.001**
Distance3-2	85.829959***	2.847164	30.145773	** < 0.001**
Interaction	− 14.129390***	2.325002	− 6.077152	** < 0.001**
Face mask	1.981377	2.324897	0.852243	0.394
Covid live risk	10.385111**	4.942763	2.101074	**0.036**
Own infection	10.160805**	4.930929	2.060627	**0.039**
Risk other c	6.372543*	3.305270	1.927995	0.054
Distance2-1*Interaction	− 11.579402**	5.689812	− 2.035112	**0.042**
Distance3-2*Interaction	− 6.183128	5.694422	− 1.085822	0.278
Distance2-1*Face mask	4.571759	5.689886	0.803489	0.422
Distance3-2*Face mask	− 1.152180	5.694036	− 0.202349	0.840
Interaction*Face mask	− 7.356908	4.649828	− 1.582189	0.114
Distance2-1*Interaction*Face mask	− 11.458153	11.379849	− 1.006881	0.314
Distance3-2*Interaction*Face mask	11.397314	11.387947	1.000823	0.317
Random effects				
σ2	550.407747			
τ00 id	278.359631			
τ00 Stimulus	55.028088			
ICC	0.377223			
N id	58			
N Stimulus	48			
Observations	2693			
Marginal R2/conditional R2	0.748/0.843			

Note. Model: Estimation.c ~ Distance * Interaction * Face mask + Covid_live_risk + Own_infection + Risk_other.c + (1|id) + (1|Stimulus)* p < 0.1 ** p < 0.05 *** p < 0.01.

Significant values are in [bold].

### ANOVA results

To examine the results in a more fine-grained way, a 3 (distance: 50 cm, 90 cm, 150 cm) × 2 (social interaction: no interaction, handshake) × 2 (face mask: with face mask, no face mask) repeated-measures ANOVA was conducted with Holmes-corrected post-hoc tests in JASP statistics (version 0.16.3.0). We used the difference in distance estimation as the dependent variable. As Mauchly’s test of Sphericity indicated that sphericity was violated in the data, Greenhouse–Geisser correction was applied. Greenhouse–Geisser corrected significant comparisons were the following: We found a significant main effect of distance (*F*(1.231, 72.648) = 41.066, *p* < 0.001, *η*^*2*^ = 0.239), an interaction effect of distance and social interaction (*F*(1.179, 69.558) = 15.857, *p* < 0.001, *η*^*2*^ = 0.030), an interaction effect of distance and face mask *F*(1.516, 89.445) = 4.296, *p* < 0.05, *η*^*2*^ = 0.002), and a triple interaction of distance, social interaction and face mask ((*F*(1.715, 101.207) = 6.934, *p* < 0.05, *η*^*2*^ = 0.005).

Post-hoc tests indicated that all comparisons within the main effect of distance reached significance (*p* < 0.05, Holmes-corrected). Further, the post-hoc tests respecting the interaction effect of distance and social interaction showed that both at a distance of 90 cm and at a distance of 150 cm (both *p* < 0.001) the effect of interaction was significant. While at a depicted distance of 90 cm, social interaction led to systematic underestimation of the distance, at a depicted distance of 150 cm, both in social interaction and in no interaction, the distance between the depicted persons was overestimated, with less overestimation when social interaction was displayed.

In addition, a post-hoc test of the interaction effect of distance and face mask (Holmes-corrected) showed that at the distance of 90 cm, wearing a face mask influenced distance estimation difference, with more underestimation when a face mask was depicted (*p* < 0.05).

**Figure 2:**
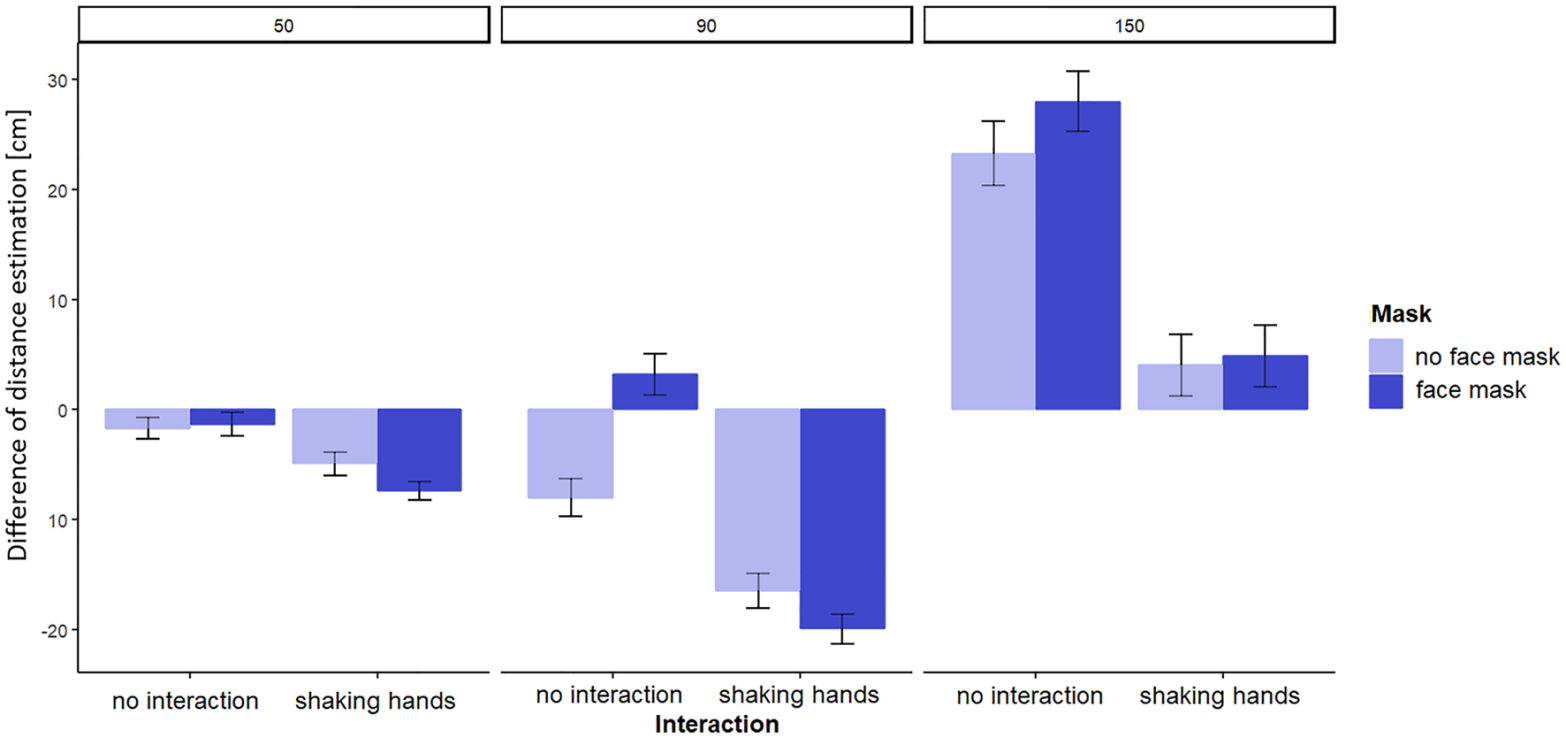
Difference of estimated real-world distance and actual distance of the pictures [cm] at a real-world distance of 50 cm, 90 cm, and 150 cm. Columns are divided into comparisons with or without social interaction (see x-axis). Light blue columns show results for estimations without face masks, dark blue bars indicate results with face masks. Vertical bars show one standard error of the mean.

Finally, a post-hoc test of the triple interaction of distance, face mask, and social interaction showed that at a distance of 90 cm without social interaction, wearing a face mask led to an overestimation of the depicted IPD. Also, at a distance of 150 cm without social interaction, wearing a face mask fostered the general overestimation of distance (both *p* < 0.05, Holmes-corrected). Thus, H3 can be partially confirmed in this analysis. Figure 2 demonstrates the results.

## Discussion

### Main variables

In this work, we aimed to examine the implicitly perceived IPD which emerged from judging interpersonal distance in a third-person perspective in the context of the COVID-19 pandemic. Crucially, we did not only examine the distance between persons but also studied the effect of displayed social interaction and face masks. Our research confirmed all three predictions.

First, without taking other factors into account, the IPD was generally underestimated when it was close to the middle distance probably reflecting avoidance behavior when depicted persons were nearer than the recommended safety distance of 1 m or more during the pandemic. This reflects a feeling of unease when a person enters the PPS of another person. Interestingly, underestimation was highest at a distance of 90 cm, where the PPS is assumed to have its borders. In addition, higher underestimation at a distance of 90 cm compared to a distance of 50 cm might be an additional indicator of awareness of the safety distance recommended by the World Health Organization: At this IPD, participants reacted especially sensitively thus highly underestimating the IPD. Here, we also need to keep in mind that the culturally accepted IPD in Germany is 90 cm. This potentially influencing factor should be addressed more carefully in future studies, involving participants from different cultural backgrounds. In contrast, at a large distance of 150 cm, participants overestimated the IPD of depicted persons. This result may suggest that participants may not have perceived any danger at a distance of 150 cm. At the same time, we cannot rule out the fact that stimuli depicting a distance of 150 cm may have been harder to estimate accurately than pictures with a smaller distance.

The repeated-measures ANOVA showed effects of social interaction at a distance of 90 cm and 150 cm. In line with our prediction, participants perceived social interaction as being dangerous in this study since social interaction was seen as dangerous or inappropriate in the context of the pandemic.

Only in the results of the repeated-measures ANOVA, we found an interaction effect of distance and face mask. In line with our expectations, at a distance of 90 cm, wearing a face mask led to more overestimation. This indicates that a face mask might have induced a feeling of safety or less danger to the participants. Nonetheless, as this effect did not reach significance in the linear mixed model analysis, our results need to be interpreted with caution.

Finally, we found that displayed face masks diminished avoidance behavior at a distance of 90 and 150 cm, but not in social interaction. From this, we conclude that face masks served as a social tool, although fear of social interaction is stronger than the perceived safety caused by the face mask. We are less afraid of being close to interacting at the border of each other’s PPS when we wear a face mask. This is in line with previous studies showing protective psychological effects of face masks^7^. However, social interaction of shaking hands was still perceived as dangerous.

It is of note, that we cannot rule out the possibility of a "distance compression effect", which refers to the tendency for individuals to underestimate the distance between others in social situations^63^. However, larger distancing (150 cm) being overestimated rather than compressed suggests that factors other than the distance compression effect may be driving participants' distance judgments in the current study, such as the use of face masks and the presence or absence of social interaction.

Taken together, these findings are generally in line with previous findings on comfortable IPD in the context of the pandemic^42–44,57^. However, our results partly contradict early findings that indicated that persons wearing face masks were perceived as more dangerous than persons without face masks^40,41^.

Our results instead describe a trend toward perceiving persons with face masks as less dangerous when they enter the PPS. The earlier studies might have found different results compared to the current study due to the timing of the research relative to the COVID-19 pandemic. Specifically, the earlier studies were conducted at the early stages of the pandemic, when face masks were still new to societies, whereas the current study was conducted at a later stage when wearing a face mask had become more normalized. This is a plausible explanation, as social norms and perceptions can change over time, particularly in response to new information or changing circumstances. As face masks became more widely adopted and promoted as a protective measure against COVID-19, individuals may have become more accustomed to seeing others wearing them and may have adjusted their behaviors and perceptions accordingly. Additionally, not engaging in physical touch such as handshakes has become increasingly acceptable instead of rude.

It's worth noting, however, that there may be other factors at play as well, such as cultural differences, individual attitudes and beliefs, and situational factors.

In general, it appears that participants in the study showed a general adoption of safety measures during the COVID-19 pandemic. Specifically, the use of face masks, which may have initially elicited feelings of danger or uncertainty, later became a means of maintaining social closeness and protecting oneself and others from the spread of the virus.

### Control variables

Two control variables reached significance in both linear mixed models: living with someone with COVID-19 risk and having had a previous own infection. These results demonstrated that both persons who lived with someone having a risk of COVID-19 and participants who had been infected rather overestimated the distance between the individuals. These results have implications for understanding the fear of COVID-19 infection within one's own household. Specifically, safety measures such as social distancing and the use of face masks may be more difficult to implement within the home environment than in social interactions outside of the home.

This is an important insight, as the home environment may be a key site of transmission for COVID-19, given that individuals spend a significant amount of time in close proximity to others within their household. Effective strategies for promoting safety within the home, such as improved ventilation, or regular cleaning and disinfecting may be critical for reducing the risk of transmission and protecting individuals and families from the spread of the virus in possible further waves of the pandemic.

Further, our results might provide a hint about the behavior of participants who already suffered from infection with COVID-19: Overestimating the distance between two persons, in general, could have caused an infection in the first place. Alternatively, persons who already suffered from infection with COVID-19 were probably not afraid of the disease anymore.

### Limitations

One limitation of the present research is that we did not explicitly ask participants which body part of the depicted individuals they used to estimate the distance to. This could be important because different body parts (e.g., face, chest, hips) may be more salient or informative cues for distance estimation, and participants may have varied in which cues they relied on. Thus, tracing participants‘ attention to either the face and thus to the face mask or away from it (e.g., by instructing them to measure the distance from chest to chest) might have changed the results.

While we did not give specific instructions on how to estimate the distance to avoid biasing participants, this lack of guidance may have introduced variability in the data and made it difficult to draw firm conclusions about which cues are most important for distance perception in the context of COVID-19.

Future research could benefit from assessing which measure point participants use to estimate distance, either through a post-questionnaire or an implicit method such as eye-tracking. This could help to clarify which cues are most important for distance perception and inform future interventions or guidelines for maintaining safe social distance during the pandemic.

Secondly, we see another possible limitation in the high exclusion rate of participants. We assume that this is connected to the first limitation: Participants who systematically underestimated depicted IPD with a mean estimated below our threshold of 50% of the closest depicted distance (i.e., 25 cm) might have tried to measure the distance between the persons’ hands, which would be zero in social interaction at 50 and 90 cm. Thus, assessing participants’ measuring points might also give insights into the reasons for the exclusion rate in our experiment. Another possible explanation is that participants did not understand the task and that instructions in future experiments need to be more specific.

Another limitation of this study is that we did not test comfortable IPD in a real-world setting but rather by showing pictures. We used this procedure because, during the pandemic, face-to-face testing bore the risk of contamination with COVID-19. Future laboratory-based studies going beyond behavioral responses would be a valuable addition to our research. In particular, functional near-infrared spectroscopy (fNIRS) proved to be an ideal tool to assess brain response to different pictures, as a recent study examining hemodynamic pattern in response to pictures of green urban areas and City Center during the COVID-19 pandemic demonstrated^64^.

Bearing in mind the limitations, it is difficult to generalize the results to the larger population, and it is important to be cautious when interpreting the findings.

## Conclusion

In this study, we tested the perceived IPD in pictures in the context of the COVID-19 pandemic. We found that participants showed avoidance behavior when the depicted persons were socially interacting at a distance of 50 or 90 cm. This effect was strongest at the border of the PPS of about 90 cm. At a wider distance of 150 cm, no avoidance behavior was found.

With caution, we interpret that at the border of the PPS, a depicted face mask helped maintain social behavior while at the farther distance of 150 cm, it fostered the feeling of not being in danger.

Our research also shows that the effect of social interaction is stronger than the effect of a face mask (if any). An explanation can be, that the safety measures of refraining from social interaction seem to be anchored in our participants’ minds while wearing a face mask is not perceived as a (crucial) safety measure.

Thus, to summarize, in this research we showed that keeping a distance is seen as protective behavior thus helping people to socialize even though they are afraid of the virus and do rather not want to physically interact with others. On the other hand, our research provides hints that wearing a face mask as well helps maintain social closeness.

Our study demonstrates the flexibility of the human proxemics behavior. With the introduction of social distancing rules, people have had to become more conscious of the distance they keep from others. This has required a shift in behavior from the natural way that people interacted before the pandemic.

Although most regions of the world have made progress in controlling the spread of COVID-19, and in pandemic-related restrictions have been lifted or relaxed, the impact of the pandemic on proxemics and other aspects of communication may persist even after the pandemic itself has subsided. For example, people may have developed new habits or preferences related to physical distancing or personal space that could continue even after the immediate threat of the pandemic has passed. Therefore, it is important for researchers to continue studying the long-term impact of the pandemic on communication and social behavior, including proxemics.

## Data Availability

The datasets and stimuli of this study are available upon reasonable request from the corresponding author.
